# Incidence of Retinal Detachment after Fellow-Performed Primary Pars Plana Vitrectomy

**DOI:** 10.1155/2013/353209

**Published:** 2013-09-19

**Authors:** Justin T. Wilkinson, Amanda B. Richards, Dongseok Choi, Joseph E. Robertson, Christina J. Flaxel

**Affiliations:** ^1^Casey Eye Institute, Department of Ophthalmology, Oregon Health & Science University, 3375 SW Terwilliger Boulevard, Portland, OR 97239-4197, USA; ^2^Private Practice in Provo, Provo, UT 84604, USA; ^3^Ophthalmology, US Air Force Joint Base Langley-Eustis, Langley, VA 23665, USA; ^4^Department of Public Health & Preventive Medicine, Oregon Health & Science University, Portland, OR 97239-4197, USA

## Abstract

*Background*. Primary pars plana vitrectomy (PPV) is often performed by vitreoretinal fellows. We aimed to evaluate the incidence of retinal breaks and detachments (RD) after fellow-performed PPV. *Methods*. We reviewed 119 consecutive cases of standard 3-port primary PPVs on 115 patients at a teaching institution from 2003 to 2006. In all cases, the primary surgeon was a vitreoretinal fellow. Patients with previous RD were excluded. Cases were reviewed for postoperative retinal detachments, and all patients were followed for up to 1 year. *Results*. Intraoperative retinal breaks occurred in 5 of 119 eyes (4.2%). One break was associated with sclerotomy and 4 were not. Postoperative RD occurred in 8 of 119 eyes (6.7%). Two RDs were sclerotomy related, 5 were not, and 1 was of indeterminate origin. *Conclusions*. Incidence of retinal breaks and RD following primary PPV by vitreoretinal fellows is low and comparable to that of fellowship-trained surgeons.

## 1. Introduction

Pars plana vitrectomy (PPV) is the most common surgery performed by vitreoretinal surgeons. This procedure is used to treat for an increasing number of indications and is done on eyes with relatively good vision. During their fellowships, vitreoretinal (VR) surgery fellows are required to perform a considerable number of these procedures and to become highly proficient. As they train vitreoretinal fellows, attending vitreoretinal surgeons are naturally concerned about safety and outcomes for their patients. Ramkissoon et al. described over 600 PPVs and found an incidence of 15% iatrogenic intraoperative breaks and a 1.7% rate of postoperative retinal detachments [1]. It is important to know how vitreoretinal fellows in training perform by comparison with these published percentages. 

Knowing whether PPV is as effective and safe when performed by vitreoretinal fellows as by attending surgeons is important for reasons besides reduced morbidity. The current United States health care climate includes growing emphasis on outcomes, and retina fellowship programs emphasize competency in surgical training. Given the frequency of retinal surgeries, it is important to determine the comparative effectiveness and safety of trainee-performed surgeries by current standards.

The purpose of this review is to evaluate the incidence of retinal breaks and retinal detachment (RD) following primary PPV performed by vitreoretinal fellows at one teaching institution and to compare those results with those in the general literature. To our knowledge, no previous studies evaluate the outcomes of primary PPV done by vitreoretinal fellows in training.

## 2. Materials and Methods

In this retrospective chart review, we reviewed medical records from 2 vitreoretinal surgeons (C. J. Flaxel and J. E. Robertson) whose patients had PPV performed by 5 vitreoretinal fellows from 2003 to 2006 at Casey Eye Institute, Oregon Health & Science University in Portland, OR, USA. This is a tertiary care referral center with an associated veterans affairs medical center (Portland VAMC). The Oregon Health & Science University Institutional Review Board gave prior approval for this study. 

For the purposes of this study, we reviewed all cases in which a vitreoretinal fellow was the primary surgeon (i.e., the fellow did the case from start to finish). Patients were either seen by the fellow in the retina fellows' clinic at the Portland VAMC or in the Casey Eye Institute Retinal Clinic as a standard retinal surgical referral.

For the review period, the fellow was the primary surgeon in 119 consecutive PPVs. The mean number of cases performed by each fellow was 23.8, the median was 18, and the range was from 4 to 52 cases. The fellows were at various stages in their 2-year fellowship training; thus, some fellows performed more cases than others with fellows at the end of their training performing more and fellows in their first year performing fewer. 

All cases were consecutive cases; at no time were any criteria used to select surgeries that would be performed by a fellow. We included no cases in which surgery was not performed by a fellow. In 48 of the 119 cases (40%), fellows operated with another fellow or with a resident. In the other 71 cases (60%), fellows operated with a fellowship-trained faculty vitreoretinal (VR) surgeon. The faculty VR surgeon stayed until the end of the case in all but 10 cases.

All cases were primary PPVs. Patients with previous RD were excluded from the study. Surgery was performed for a wide range of indications, as shown by the sampling of consecutive cases in Table 1. All surgeries were standard 20-gauge 3-port PPVs and all patients were followed for up to 1 year after surgery. The median follow-up time was 12 months, and the average follow-up time was 8.5 months.

All surgeries were performed at Casey Eye Institute in a standard fashion with a wide-angle viewing system, utilizing either the AVI (Advanced Visual Instruments, New York, NY, USA) or BIOM (Oculus, Lynnwood, WA) lens system. Operative reports and preoperative and postoperative data were evaluated for all 119 eyes (115 patients) to determine the incidence of retinal breaks and RDs and any potentially associated risk factors for up to 1 year after surgery. We calculated this incidence using a rate of 0.4 sclerotomy-related retinal breaks or detachments per 100 eye-months of followup. 

## 3. Results and Discussion 

The study included 119 eyes of 115 patients. There were 68 right eyes and 51 left eyes. Patients were 32 women and 87 men.

Intraoperative retinal breaks occurred in 5 of the 119 operated eyes (4.2%). One break was associated with a sclerotomy site; the other 4 were not associated with sclerotomy sites.

**Figure 1:**
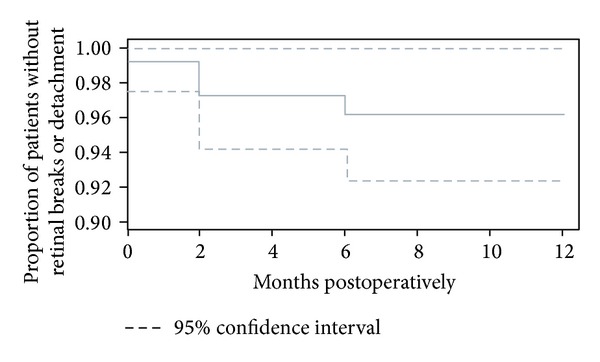
Retinal breaks or detachments after pars plana vitrectomy by vitreoretinal fellows. The figure shows the proportion of retinal breaks or detachments in 119 eyes of 115 patients undergoing pars plana vitrectomy with a vitreoretinal fellow as the primary surgeon. Patients were followed for up to 1 year after surgery.

There were 8 postoperative retinal detachments (6.7%). Two of these were sclerotomy related, 5 were not sclerotomy related, and 1 was of indeterminate origin. As noted before, this equates to 0.4 sclerotomy-related retinal breaks or detachments per 100 eye-months of followup, with a median follow-up of 12 months (Figure 1). Seven of the 8 postoperative RDs developed within 2 months after surgery (87.5%) and 1 of the 8 (12.5%) within 6 months postoperatively. Three of 8 (37.5%) RDs occurred in eyes with macular holes and 2 of 8 (25%) in eyes with retained lens fragments. Of the remaining 3 RDs, 1 patient was post trauma, 1 had a nonclearing vitreous hemorrhage secondary to proliferative diabetic retinopathy, and 1 had a subretinal hemorrhage secondary to neovascular AMD. 

Since the advent of PPV, it has been recognized that this surgery is associated with significant risk of complications, including cataract formation, increased intraocular pressure, vitreous hemorrhage, endophthalmitis, severe intraocular inflammation, sclerotomy-related complications, retinal tears, retinal detachment, and complications arising from the use of silicone oils, intraoperative dyes such as indocyanine green (ICG), and the use of perfluorocarbon liquids [1–15]. 

Despite advances in vitreoretinal surgery in recent years, including the advent of small-gauge cannulated systems, retinal tears at the time of surgery are still a major complication, even in the hands of experienced vitreoretinal surgeons [1, 6, 9]. Retinal detachment is also recognized to occur as a result of excessive wound healing at the incision site; thus, it can occur at any time after the original PPV [12, 13]. Even though many cases are now being done with small gauge, a recent survey by Market Scope shows that 68% of retina specialists still utilized 20-gauge surgery [14].

A recent paper by Ramkissoon et al. describing over 600 PPVs found an incidence of 15% iatrogenic intraoperative breaks and a 1.7% rate of postoperative retinal detachment [1]. The authors concluded that breaks arise from traction at entry sites as well as from intraocular manipulation. Thus, some of these complications might be expected to happen more often in cases performed by trainees. In our series, we did not find such a high incidence of retinal breaks. However, the rate of retinal detachment was higher, which could mean that some breaks that would have later led to retinal detachment were present at the time of initial surgery but were not seen. 

Many other case series describe the frequency of iatrogenic retinal breaks and postoperative retinal detachment in the literature [2–4, 6–11]. Rates of these complications in macular hole surgery vary from 0% to 14.6%, with retinal detachment occurring in 1.1% to 14% [1–3, 8, 9]. Reported rates for epiretinal membrane surgery range from 0.6% to 6.9% for peripheral retinal breaks and from 1.4% to 6% for retinal detachment [2–13, 15]. 

In a 2007 report, Wimpissinger and Binder reported an incidence of 0% intraoperative breaks [15]. However, they saw a postoperative retinal detachment rate of 4.5% in a series similar to ours, with a variety of indications for PPV [15]. They attributed the retinal detachments to sclerotomy-related breaks, which could mean that at least some of these breaks were present at the end of the procedure but were not noted, as we have postulated for some of the retinal detachments in our series [15].

To our knowledge, there have been no studies evaluating the outcomes of these surgeries when performed by vitreoretinal fellows in training, though the study by Territo et al. does include cases performed at least partly by fellows and residents [9]. It is possible that other studies include cases performed by fellows, although we would expect authors to note if fellows did the majority of PPVs.

In contrast to VR surgery, numerous studies have evaluated the outcomes of other surgeries performed by trainees, such as resident-performed cataract surgery and trabeculectomy and endothelial keratoplasty performed by cornea fellows [16–20]. One might expect a relatively poorer outcome in fellow-performed surgeries versus those performed by attending physicians. However, in a study of cornea fellows, Chen et al. found excellent surgical outcomes for fellows compared with their attending physicians, as did prior studies of fellow-performed corneal transplants [20]. In our current series of cases performed by vitreoretinal fellows, we had an overall rate of intraoperative breaks of 4.2%, which compares very well to the published rates from 0% to 24% [1–9, 15] for experienced vitreoretinal surgeons, and a rate of retinal detachment of 6.7%, which is also comparable to the previously reported rates ranging from 0% to 15.8% [2–6]. It is also important to note that the fellows in our study operated on consecutive cases, so the case mix was the same as that experienced VR surgeons would encounter at our tertiary care referral center (Table 1). 

## 4. Conclusions 

As with the other outcome evaluations, this evaluation can serve at least 2 purposes: (1) to determine the current quality of training at representative ophthalmology programs for teaching purposes and (2) to reassure patients that surgical results are being evaluated, published, and discussed in an open forum [17]. Our results suggest that the incidence of retinal breaks and RD following primary PPV performed by vitreoretinal fellows in an academic setting is low and comparable with the incidence reported among all fellowship-trained surgeons in previous studies in the literature. 

## Figures and Tables

**Table 1:** Indications for pars plana vitrectomy performed by vitreoretinal fellows at Casey Eye Institute, Oregon Health & Science University.

Indications for PPV	
Nonclearing vitreous hemorrhage (VH) status after ruptured globe	
Intraocular foreign body (IOFB)	
Nonclearing VH, status after blunt trauma	
Endophthalmitis following ruptured globe	
Postoperative endophthalmitis	
Chronic uveitis versus endophthalmitis	
Retained lens fragments	
Epiretinal membrane	
Macular hole	
Dislocated intraocular lens	
VH from proliferative diabetic retinopathy	
VH from central retinal vein occlusion	
VH from neovascular age-related macular degeneration	
Vitreomacular traction	
Cataract following iridocyclectomy for iris/ciliary body melanoma	
Aqueous misdirection syndrome	
